# Effects of Water Models on Binding Affinity: Evidence from All-Atom Simulation of Binding of Tamiflu to A/H5N1 Neuraminidase

**DOI:** 10.1155/2014/536084

**Published:** 2014-02-02

**Authors:** Trang Truc Nguyen, Man Hoang Viet, Mai Suan Li

**Affiliations:** ^1^Institute for Computational Science and Technology, Quarter 6, Linh Trung Ward, Thu Duc District, Ho Chi Minh City, Vietnam; ^2^Institute of Physics, Polish Academy of Sciences, Aleja Lotnikow 32/46, 02-668 Warsaw, Poland

## Abstract

The influence of water models SPC, SPC/E, TIP3P, and TIP4P on ligand binding affinity is examined by calculating the binding free energy Δ*G*_bind_ of oseltamivir carboxylate (Tamiflu) to the wild type of glycoprotein neuraminidase from the pandemic A/H5N1 virus. Δ*G*_bind_ is estimated by the Molecular Mechanic-Poisson Boltzmann Surface Area method and all-atom simulations with different combinations of these aqueous models and four force fields AMBER99SB, CHARMM27, GROMOS96 43a1, and OPLS-AA/L. It is shown that there is no correlation between the binding free energy and the water density in the binding pocket in CHARMM. However, for three remaining force fields Δ*G*_bind_ decays with increase of water density. SPC/E provides the lowest binding free energy for any force field, while the water effect is the most pronounced in CHARMM. In agreement with the popular GROMACS recommendation, the binding score obtained by combinations of AMBER-TIP3P, OPLS-TIP4P, and GROMOS-SPC is the most relevant to the experiments. For wild-type neuraminidase we have found that SPC is more suitable for CHARMM than TIP3P recommended by GROMACS for studying ligand binding. However, our study for three of its mutants reveals that TIP3P is presumably the best choice for CHARMM.

## 1. Introduction

The determination of binding affinity is a central problem in computer-aided drug design which is a useful tool to search for potential leads for various diseases. The accuracy of estimation of the binding free energy Δ*G*_bind_ of ligand to receptor depends on computational methods and modeling of receptor-ligand complex. The docking method is usually used for locating binding sites and virtual screening of potential drug candidates from large data bases. This approach suffers from low accuracy and its results usually have to be refined by more sophisticated methods based on the molecular dynamics (MD) simulation. In many cases MD methods can reproduce reliable results on binding free energy having acceptable correlation with experimental data [1–5]. Among them Molecular Mechanic-Poisson Boltzmann Surface Area (MM-PBSA) [1], thermodynamic integration (TI) [2], linear interaction energy (LIE) [3], linear response approximation (LRA) [6, 7], free energy perturbation (FEP) [4], and steered MD [8, 9] methods are widely used. Each method should be considered carefully to compromise between CPU time efficiency and accuracy level.

In modeling of biosystems in aqueous environment, it is important to develop appropriate force fields and water models. Force fields, which are given in the form of empirical potential energy functions, have been developed by different groups. Today OPLS, CHARMM, AMBER, and GROMOS are the most popular force fields for all-atom simulation of biomolecules. To describe aqueous environment, one can use various models such as SPC [10], SPC/E [11], TIP3P [12], and TIP4P [13]. The adjustment of parameters of these models is based on their ability to reproduce the enthalpy of vaporization and the density of water. SPC/E is especially accurate for capturing experimental properties of water such as the diffusion coefficient and dielectric constant. Because SPC, SPC/E, TIP3P, and TIP4P are relatively simple and able to provide reasonable results, they are often employed for simulation of peptides [14, 15] and proteins [16].

Previous studies [17, 18] have revealed that different force fields provide different estimations for Δ*G*_bind_. Recently, the role of water molecules in the binding process has been considered [19–21]. GROMACS manual (http://www.gromacs.org/Support/Online_Manual) suggests that for all-atom simulation of biomolecules water model TIP3P is suitable for AMBER and CHARMM, while TIP4P and SPC are more appropriate, respectively, for OPLS and GROMOS force fields. However, what water model is the best fit for a given force field in computation of Δ*G*_bind_ remains largely unknown.

To shed light on this problem, in this paper we study the impact of combinations of four main water models SPC [10], SPC/E [11], TIP3P [12], and TIP4P [13] with AMBER99SB [24], CHARMM27 [25], OPLS-AA [26], and GROMOS96 43a1 [27] force fields on the binding affinity of Tamiflu to the wild type (WT) of A/H5N1 neuraminidase (NA). We have chosen the NA-Tamiflu complex because A/H5N1 virus causes great damage to live poultry markets [28], especially being recognized as human transmitted virus [29]. More importantly, the binding free energy of Tamiflu to NA has been experimentally determined [23] and this gives us the opportunity to compare theoretical estimates with the experimental ones.

Using MM-PBSA method we have shown that combinations AMBER-TIP3P, OPLS-TIP4P, and GROMOS-SPC are the best choice for estimation of Δ*G*_bind_ of Tamiflu. This result is in agreement with the GROMACS recommendation, which is followed from force field development [24–27]. Contrary to the GROMACS suggestion, it is shown that SPC is more suitable for CHARMM than TIP3P but this conclusion is valid for the wild type of NA. Our study of three mutants H274Y, N294S, and Y252H reveals that TIP3P is presumably the best choice for CHARMM as suggested by GROMACS.

It is found that Δ*G*_bind_ obtained by the OPLS force field is much less sensitive to water models compared to other force fields. The difference in water models seems to have the drastic effect in CHARMM modulating both the receptor-ligand interaction energy and hydrogen bond network in binding area. For all studied force fields, SPC/E is worse than other aqueous models overestimating Δ*G*_bind_.

## 2. Materials and Methods

### 2.1. Crystal Structure of A/H5N1 NA and Parametrization of Tamiflu

The initial structures of A/H5N1 WT and mutants H274Y and N294S were obtained from Protein Data Bank with PDB ID 2HU4, 3CL0, and 3CL2, respectively [23]. Y252H was derived by the corresponding mutation in WT structure using the mutagenesis module, integrated in PyMOL package [30]. For Tamiflu we use the oseltamivir carboxylate type. Its charges and atom types, used for MD simulation, are described in detail in our previous work [18]. Namely, for united-atom GROMOS96 43a1 force field, charges and atom types of oseltamivir were fully parametrized by Dundee PRODRG2.5 Server (Beta) [31]. For the remaining all-atom force fields, atomic partial charges for Tamiflu were derived by ESP charge. To obtain optimal geometry for electrostatic potential calculations, its structure is first optimized with the help of Gaussian98 [32] using the B3LYP/6-31G* level of theory. Fitting charges to the electrostatic potential was subsequently done by the RESP method. Atom types for Tamiflu were derived from different modules to get along with each force field. ACPYPE [33] and MKTOP [34] were adjusted to provide suitable atom types in OPLS-AA/L [26], while for AMBER99SB and CHARMM27 [25], atom types were named by ACPYPE and SwissParam [35], respectively.

### 2.2. Water Models

**Figure 1:**
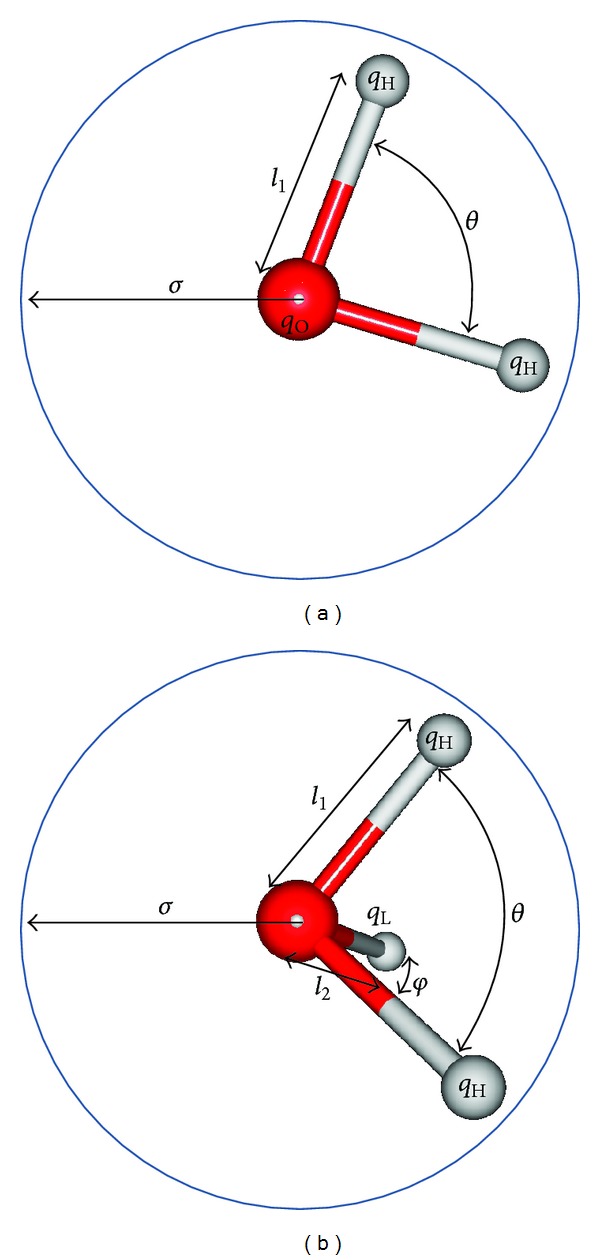
The 3-site (a) and 4-site (b) water models [22]. The labels are explained in Table 1.

Water, known as an indispensable solvent in almost chemical and biological reactions, has been built in different ways to obtain reasonable models for computational study [10–13]. A water molecule is characterized by its geometrical parameters such as bond lengths and angles which could be kept rigid or flexible during simulation. Each model is parametrized with atomic partial charges of oxygen and hydrogen and assigned with dispersion and repulsion forces approximated by Lennard-Jones potential [22]. Water models are categorized by the number of points used to shape them, by rigid or flexible structures, and by integration or not with polarization effects [22]. There are 46 distinct models [36] that have from 3 to 6 sites. However, only 3- and 4-site models (Figure 1) are often used in simulations of biological systems. For 3-site model like SPC [10] and TIP3P [12], a water molecule is constructed by one oxygen atom and two hydrogen atoms. Each atom is assigned with atomic partial charge, but only oxygen is allowed to have the Lennard-Jones interaction with other atoms. The van der Waals (vdW) interaction among hydrogen atoms was not parametrized yet. Three-site models are known as rigid and have the experimental geometry of water, except SPC which has the ideal tetrahedral angle of water as 109.47, but not 104.5°. SPC/E [11], an updated version of SPC model, adds an average polarization correction to the potential energy function, resulting in the better density and diffusion constant than SPC model.

The four-site model TIP4P [13] is a rigid planar four-site interaction potential for water (Figure 1), having a similar geometry to the Bernal and Fowler model [37]. Here the negative charge is shifted from the oxygen atom to a point 0.15 Å  along the bisector between hydrogen atoms. In this paper, we just limit our study to only four frequently used water models SPC, SPC/E, TIP3P, and TIP4P (Figure 1). Their geometrical and physical characteristics are described in Table 1. Here *q*_H_, *q*_O_, and *q*_L_ are the partial charges of hydrogen, oxygen, and lone pair, respectively. *θ* and *ϕ* are the H–O–H and lone pair-O–H angles, while *ɛ* and *σ* are the well depth and vdW radius, respectively.

### 2.3. Molecular Dynamic Simulations

Complex of NA-Tamiflu is placed in a triclinic box of around 12000 water molecules with 1 nm distance between the solute and box (a typical snapshot is shown in Figure S1 of supplementary material available online at http://dx.doi.org/10.1155/2014/536084). The receptor and ligand have 3832 and 5749 atoms in the united atom and all-atom models, respectively. Periodic boundary condition is imposed in three directions. We use 1.4 nm and 1.0 nm cut-off for vdW and electrostatic interactions, respectively. Long range electrostatic interaction was computed by the particle-mesh Ewald summation method [38]. Equations of motion were integrated using a leap-frog algorithm [39] with a time step 1 fs. The nonbonded interaction pairlist was updated every 10 fs with the cut-off of 1 nm. All systems were neutralized by adding counter-ions and then minimized to remove the local strain in protein upon addition of all hydrogen atoms and to remove bad vdW contacts with water. By using the conjugate gradient method for every 50 steps of steepest descent, minimization is converged when maximum force becomes smaller than 0.01 kJ/mol/nm. Then, all bonds of protein were restrained, leaving remaining parts to relax for 100 ps to obtain evenly distributed system. The temperature was gradually heated to 300 K during 100 ps with 5 kcal/mol harmonic restraints in all systems. The equilibration was next performed, coupled with temperature and pressure. Constant temperature 300 K was enforced using Berendsen algorithm [40] under 50 ps NVT simulation with a damping coefficient of 0.1 ps. Parrinello-Rahman pressure coupling [41] was used in 100 ps NPT run for 1 atm with the damping coefficient of 0.5 ps. Final NPT simulations of 20 ns were carried out for all force fields. Each force field is combined with four different water models SPC, SPC/E, TIP3P, and TIP4P, except GROMOS, which uses only SPC and SPC/E models. In total, we have 14 different models for the NA-Tamiflu complex. All simulations have been carried out in the GROMACS suit with Gromacs-4.5 package [42].

### 2.4. Binding Free Energy Calculation by MM-PBSA

The details of MM-PBSA method are given in our previous work [18]. Overall, in this method the binding free energy of ligand to receptor is defined as follows:

(1) $$\begin{array}{c} \Delta G_{\text{bind}}=\Delta E_{\text{elec}}+\Delta E_{\text{vdw}}+\Delta G_{\text{sur}}+\Delta G_{\text{PB}}-T\Delta S, \end{array}$$

where Δ*E*_elec_ and Δ*E*_vdw_ are contributions from electrostatic and vdW interactions, respectively. Δ*G*_sur_ and Δ*G*_PB_ are nonpolar and polar solvation energies. The entropic contribution *T*Δ*S* is estimated using the normal mode approximation [18]. From 20 ns MD simulation output only snapshots collected in equilibrium are used to compute the binding free energy given by (1).

### 2.5. Measures Used in Data Analysis

The C_*α*_ root-mean-square deviation (RMSD) is employed to measure the deviation of receptor structures from the initial configuration. The hydrogen bond (HB) is assumed to be formed if the distance between proton donor (D) and proton acceptor (A) is less than 0.35 nm and the angle H-D-A is also less than 30°.

### 2.6. Definition of Binding Site

**Figure 2:**
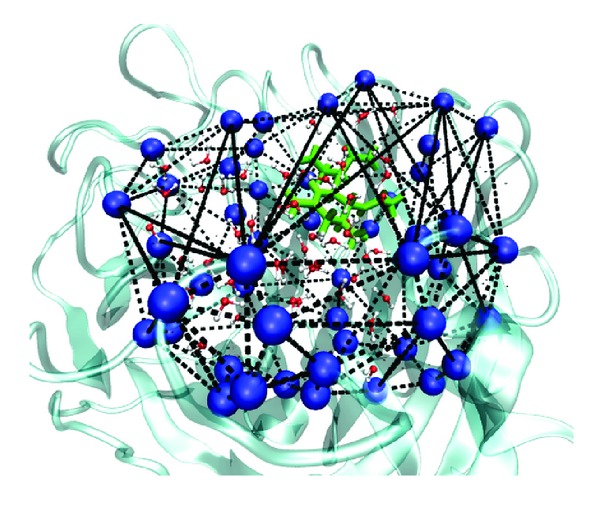
The construction of convex hull for the binding site. C_*α*_ atoms of fifty residues which define the binding pocket are shown in blue ball. Not all sides of polyhedron are shown. Oseltamivir is colored in green. Water molecules are also presented.

The binding pocket is defined as a space surrounded by 50 amino acids as shown in Figure S2. Our definition is compatible with that of Cheng et al. [43]. The list of these amino acids is given in Table 2. Volume of the binding pocket is approximately estimated as volume of smallest convex hull which contains all of the fifty C_*α*_ atoms [44–46] (Figure 2). The number of water molecules inside the convex hull is considered as the number of water molecules in the binding site. The binding pocket volume and number of water molecules inside it are calculated by Matlab software [47]. The water density in binding site is a ratio of the number of water molecules to its volume.

## 3. Results and Discussion

In this section we present results obtained for WT NA if not otherwise stated.

### 3.1. Equilibration Time Scales Depend on Force Field and Water Model

**Figure 3:**
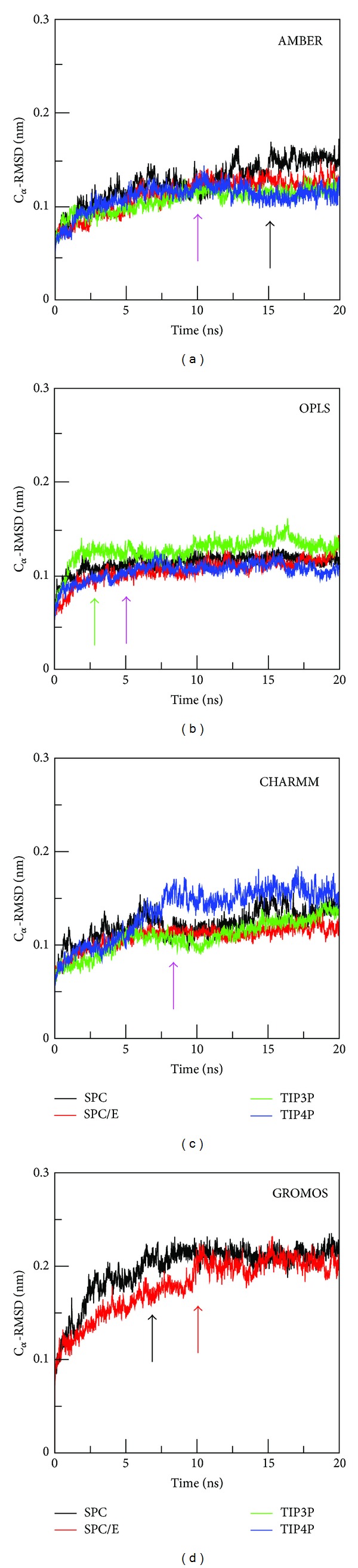
C_*α*_-RMSD of wild-type NA when interacting with Tamiflu during 20 ns simulations with different combination of force fields and water models. For AMBER equilibration time *t*_eq_ ≈ 15 ns for SPC (black arrow) while *t*_eq_ ≈ 10 ns for remaining water models (magenta arrow). In OPLS *t*_eq_ ≈ 3 ns for TIP3P (green arrow) and 5 ns for other models (magenta arrow). In the CHARMM case all systems reach equilibrium after about 8 ns. For GROMOS *t*_eq_ ≈ 7 ns (black arrow) and 10 ns (red arrow) for SPC and SPC/E, respectively.

The time dependence of C_*α*_-RMSD of NA in complex with Tamiflu is shown in Figure 3 for different sets of force fields and water models. The equilibration of system is reached when RMSD gets saturation. In AMBER the equilibration time *t*_eq_ ≈ 15 ns for SPC, while *≈*10 ns is needed to equilibrate the system in other water models (Figure 3). SPC gives the largest RMSD in equilibrium state. In OPLS RMSD reaches saturation quite fast, after about 3 ns for TIP3P and 5 ns for remaining models (Figure 3). TIP3P provides a bit larger departure from the initial structure compared to remaining models.

For CHARMM *t*_eq_ ≈ 8 ns for all sets (Figure 3). The effect of water on stability of the NA-Tamiflu complex is at most pronounced in CHARMM, where TIP4P affects the stability to a greater extent than other models. In GROMOS one has different time scales for equilibration in SPC (*t*_eq_ ≈ 7 ns) and SPC/E (*t*_eq_ ≈ 10 ns), but in equilibrium the average values of RMSD are almost the same for both water models. Due to united-atom nature GROMOS is the most unstable having average value of RMSD ≈0.2 nm against 0.13 nm of other force fields (Figure 3).

### 3.2. Estimation of Binding Free Energy by MM-PBSA Method

#### 3.2.1. Effect of Water Model on the Receptor-Ligand Interaction Energy

**Figure 4:**
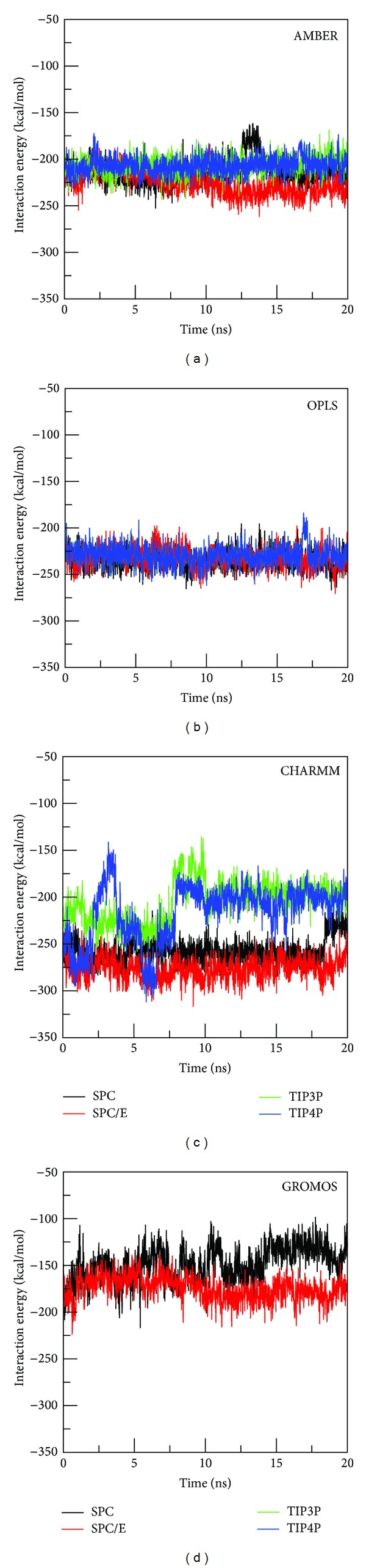
Time dependence of interaction energies of wild-type NA with Tamiflu during 20 ns simulation with different combination of force fields and water models.

The interaction energy *E*_int⁡_ between ligand and receptor is shown in Figure 4. The most pronounced dependence on water is observed for CHARMM as *E*_int⁡_ fluctuates at very different levels. Particularly, TIP3P and TIP4P models make the interaction energy highly unstable during the first 7 ns, while it remains almost stable during the whole MD run for SPC and SPC/E (Figure 4). In equilibrium TIP3P and TIP4P give lower interaction energy than SPC and SPC/E. Average interaction energy ${\bar{E}}_{\mathrm{int}}\approx -259.2$, −279.7, −228.5, and −216.9 kcal/mol for SPC, SPC/E, TIP3P, and TP4P, respectively.

In GROMOS *E*_int⁡_, obtained by using SPC, is higher than SPC/E. The effect of water modeling is also visible for AMBER, where SPC/E provides the lowest interaction energy in equilibrium. *E*_int⁡_ is almost the same in TIP3P and TIP4P (Figure 4). As in the RMSD case (Figure 3), the results, obtained by the OPLS force field, are not affected much by water models (Figure 4). The combination of GROMOS with SPC and SPC/E gives the highest receptor-ligand interaction energy among four force fields, while the lowest *E*_int⁡_ is obtained by CHARMM-SPC and CHARMM-SPC/E.

#### 3.2.2. Electrostatic Interaction Dominates over vdW Interaction in All Models

The separate contributions of these two interactions are shown in Figures S3 and S4. Clearly, the electrostatic interaction is far superior than vdW in binding affinity of Tamiflu to NA. This observation was reported previously [18] for a few number of models, but the role of water has not been explored yet.

As follows from Tables 3–6, the contribution of vdW interaction to the binding free energy is not sensitive to water models for all force fields except CHARMM where SPC makes markedly higher contribution compared to other models. The effect of environment on the electrostatic interaction is weak in OPLS (Table 4) leaving Δ*E*_elec_ almost equal in 4 water models. For AMBER (Table 3) SPC/E gives the lowest estimation for Δ*E*_elec_, while the drastic water effect is observed in CHARMM (Table 5) and GROMOS (Table 6). In the latter case two models yield the difference in Δ*E*_elec_ of about 30 kcal/mol, but SPC/E and TIP4P in CHARMM provide even larger difference of *≈*61.5 kcal/mol.

#### 3.2.3. Binding Free Energy Depends on Water Models

Apolar solvation energy Δ*G*_sur_ and entropy contributions are not sensitive to force fields and water models (Tables 3–6). Δ*G*_sur_ is about 4.5 kcal/mol, while −*T*Δ*S* is 13–15 kcal/mol for all models. The dependence of Δ*G*_bind_ on water mainly comes from competition between the electrostatic energy and polar solvation energy Δ*G*_PB_. If they compensate each other as in the case of AMBER and GROMOS, then the absolute value of Δ*G*_bind_ is small (Tables 3 and 6). For OPLS Δ*G*_PB_ is far below the absolute value of Δ*E*_elec_ leading to large Δ*G*_bind_. This result suggests that the charge parametrization of OPLS is not suitable for studying binding affinity of oseltamivir to NA and its mutations [18]. Since overestimation of Δ*G*_bind_ was obtained by the MM-PBSA method, it remains unclear whether other methods would change this conclusion. The similar noncompensation effect is observed in CHARMM-SPC/E set (Table 5) where Δ*G*_bind_ is also far below the experimental result (*≈*−40.85 kcal/mol).

SPC/E generates the most negative values for both electrostatic and vdW interactions compared with other 3 models (Figure 4). Therefore, this model provides the highest binding affinity in all studied force fields (Tables 3–6). This observation agrees with the previous study of Hu and Jiang [16] that the Coulomb interaction between water and lysozyme is more negative in SPC/E than in SPC and TIP3P since SPC/E has weaker self-diffusion than others, but closer to the experiment. In GROMOS force fields SPC and SPC/E give Δ*G*_bind_ ≈ −11.79 and −18.56 kcal/mol, respectively (Table 6). Clearly, SPC result is closer to the experiment [23].

Averaging the binding free energy over water models, one has ${\bar{\Delta G}}_{\text{bind}}=-18.36\pm 4.16$, −66.96 ± 2.26, −28.33 ± 8.62, and −15.18 ± 4.79 for AMBER, OPLS, CHARMM, and GROMOS, respectively. Thus, the strongest effect of water modeling is observed in CHARMM as the departure from the average value ${\bar{\Delta G}}_{\text{bind}}$ is about 8.6 kcal/mol, while the weakest influence is seen in OPLS with deviation of *≈*2.3 kcal/mol.

### 3.3. Recommendation for the Best Sets of Force Field and Water Model

To recommend the best combination one has to rely on the experimental results. The experiments of Collin's group [23] have shown that the binding free energy of Oseltaminir to A/H5N1 NA Δ*G*_bind_ = −13.12 kcal/mol. Clearly, in AMBER TIP3P is the best fit to the experiments giving Δ*G*_bind_ = −13.91 kcal/mol (Table 3). Thus, in accord with the GROMACS recommendation, AMBER-TIP3P is the best choice for studying ligand binding affinity. The agreement with the GROMACS's suggestion has been also obtained for GROMOS-SPC and OPLS-TIP4P (Tables 6 and 4) having Δ*G*_bind_ closer to the experiments than other sets. It should be noted that OPLS-TIP4P is marginally better than OPLS-TIP3P because their difference in Δ*G*_bind_ is less than 1 kcal/mol. So OPLS-TIP3P may not be a bad choice for estimation of binding affinity.

For CHARMM the closest to experiment result (Δ*G*_bind_ = −17.79 kcal/mol) falls into SPC model (Table 5). TIP3P is ranked second having Δ*G*_bind_ = −23.62 kcal/mol which is far from the experimental estimate. Thus, based on the results obtained for WT NA, one may recommend to use CHARMM-SPC instead of CHARMM-TIP3P suggested by the GROMACS. Since this conclusion is obtained for one system, to ascertain that SPC is the best choice for CHARMM we have computed Δ*G*_bind_ for three more systems including mutants Y252H, N294S, and H274Y which have been studied experimentally [23]. We summarize the main results in Table 7 providing details of calculations for different water models in Supporting Information (SI) (Figure S5 and Tables S1–S4). The experiments show the ranking for binding affinity as Y252H > WT > N294S > H274Y. This ranking is correctly captured by TIP3P and TIP4P but not SPC as well as SPC/E (Table 7). Comparing absolute values of Δ*G*_bind_ with the experiments one can see that SPC and SPC/E are the best for WT and Y252H, while TIP3P is the best for both N294S and H274Y. Taken together, in accord with GROMACS's suggestion, TIP3P is most suitable for CHARMM.

### 3.4. Hydrogen Bond Network at the Binding Site

From previous MM-PBSA results, the hydrogen bonding, which mostly contributes to the electrostatic energy, plays the key role in the interaction between Tamiflu and A/H5N1 NA [18, 48]. However, the role of water has not been explored yet. Figure S6 shows the time dependence of HBs obtained by different force fields and water models. The HB number not only levels significantly among force fields but also depends on aqueous environments.

#### 3.4.1. Amber Force Field

**Figure 5:**
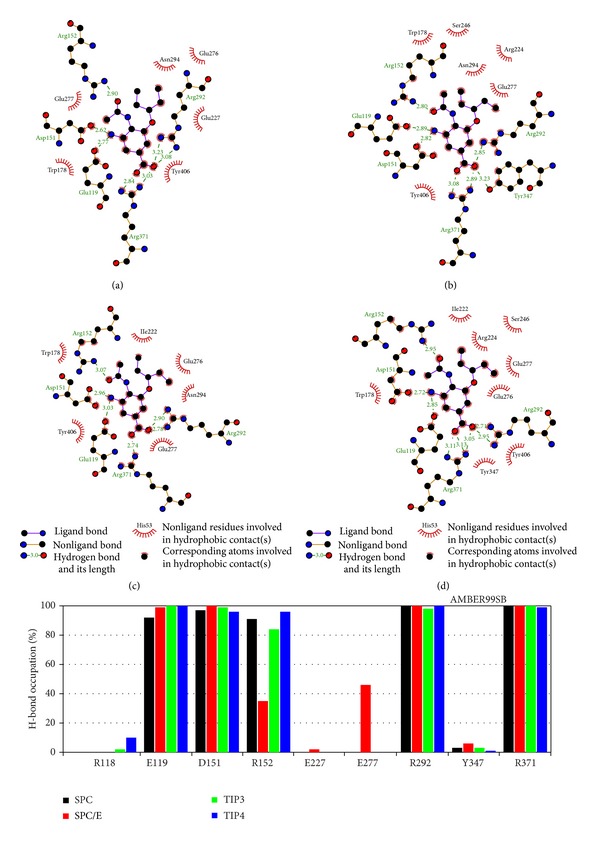
Typical snapshots for hydrogen bond network between Tamiflu's charged groups and residues of NA at the binding site obtained by AMBER99SB force field with SPC (a), SPC/E (b), TIP3P (c), and TIP4P (d). Oseltamivir is hydrobonded with –COO^−^ and –NH_2_ (R371, R292); –OH (Y347); −NH_3_^+^ and –COO^−^ (D151, E119); NHAc and −NH_2_ (R152) of NA. All hydrogen atoms are implicit. The lower panel refers to the probability of formation of HBs between ligand and receptor. The results are averaged over the last 2 ns of simulation. Black, red, green, and blue refer to SPC, SPC/E, TIP3P, and TIP4P, respectively.

Typical HB networks of four sets with AMBER are shown in Figure 5, where one has 7, 7, 6, and 7 HBs for SPC, SPC/E, TIP3P, and TIP4P, respectively. For all water models oseltamivir has the strong hydrogen bonding with residues E119, D151, R292, and R371 (lower panel of Figure 5). Within SPC/E the H-bonding with R152 is weaker than other models which have the population exceeding 80%. The strong interaction with E277 is observed only for this water model. Thus, in terms of individual contributions of ligand atoms SPC/E differs from other models. However, in equilibrium the average numbers of HBs are almost equal in all aqueous environments having $\bar{\text{HB}}(t)\approx 6.6$, 6.7, 7.0, and 7.0 for SPC, SPC/E, TIP3P, and TIP4P, respectively (Figure S6).

#### 3.4.2. OPLS Force Filed

For OPLS four aqueous models show nearly the same HB networks (Figure S6) (in equilibrium $\bar{\text{HB}}(t)\approx 6.2$, 6.0, 6.0, and 7.2 for SPC, SPC/E, TIP3P, and TIP4P, resp.). This is not surprising because they also have little effect on the binding free energy as discussed in the previous section (Table 4). HB patterns are quite similar among various water models in AMBER and OPLS force fields (Figure 5 and Figure S7) implying that the geometry of ligand and area around the binding pocket does not depend much on water models. A slight difference is in population at residues R152 and E277 for two force fields.

#### 3.4.3. CHARMM Force Filed

The situation becomes very different in the case of CHARMM where water has the strong effect on the HB network (Figure S8). The average number of HB in equilibrium $\bar{\text{HB}}(t)$ varies between 5.5 for SPC and 4.4 for TIP4P (Figure S6). Contrary to AMBER and OPLS, only R371 remains the key residue for 4 aqueous models having the population more than 75%. SPC gives also strong H-bonding with E119 and D151 (population >50%), while D151, R152, and R292 are H-bonded with Tamiflu for the most simulation time with SPC/E (Figure S8). The ligand forms HB with R118 and E277 if one uses TIP3P but not other models (Figure S8). TIP4P shows the modest HB population with E119, R152, and Y347, while together with R371 residue R292 is conserved in this water model. The diversity of HB networks in CHARMM presumably causes strong variation of the binding free energy among 4 aqueous models (Table 5).

#### 3.4.4. GROMOS Force Filed

As follows from Figure S6, due to the united-atom approximation used for this force field the number of HBs is much lower ($\bar{\text{HB}}(t)\approx 0.7$ and 1.6 for SPC and SPC/E) than other force fields. Consequently, HB networks are very poor (Figure S9). H-bonding in SPC/E is stronger than SPC leading to its higher binding affinity (Table 6). Residue R152 has the substantial population in this water model. For SPC H-bonding is weak for all residues from the binding pocket.

### 3.5. Effect of Hydration on Binding Affinity

#### 3.5.1. AMBER Force Field

**Figure 6:**
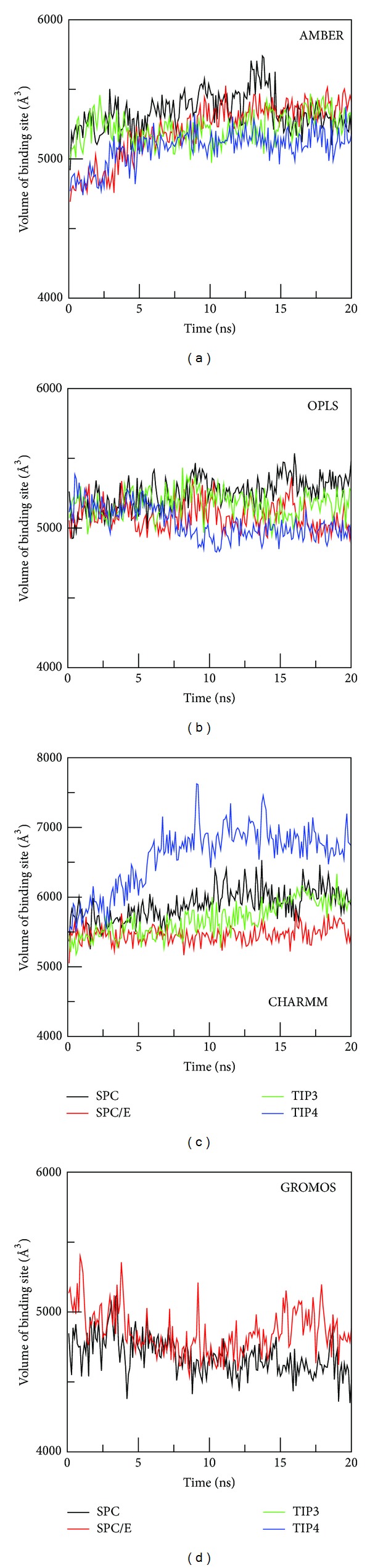
Time dependence of binding pocket volume in different force fields and water models.

**Figure 7:**
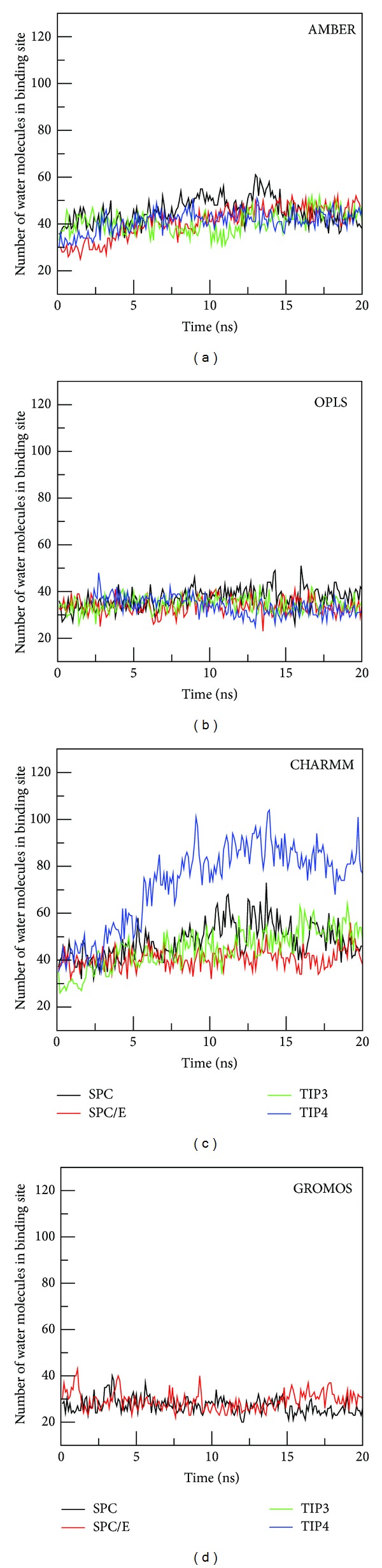
Time dependence of the number of water molecules inside the binding pocket.

Volume of the binding pocket, estimated by approximate polyhedron (Materials and Methods), fluctuates during the course of MD simulation (Figure 6) depending on types of hydration. It gets saturated in equilibrium and the average volume is shown in Table S5. The largest volume is obtained for SPC/E, while TIP4P provides the smallest volume. The time fluctuation of the number of water molecules inside the pocket (Figure 7) indicates that water molecules keep going out and coming back (see Movie 1). The weak dependence on water models is observed for AMBER force field because in equilibrium the binding pocket contains 42–46 water molecules (Table S5). SPC/E widens the binding site volume to a greater extent compared to other models providing the largest number of water molecules.

**Figure 8:**
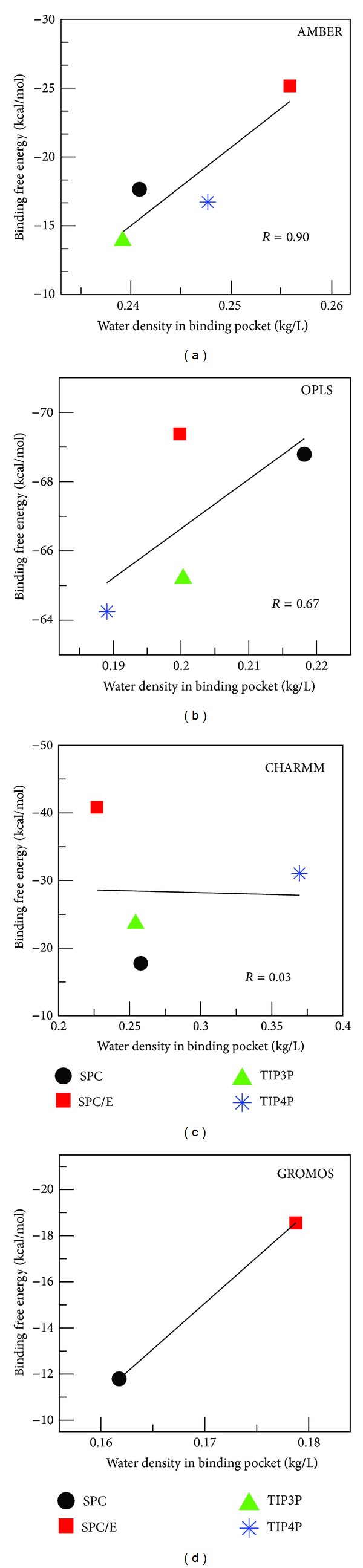
Δ*G*_bind_ versus water density at the binding site in all force fields.

Figure S10 shows the time dependence of the water density in the binding space *ρ*_w_^bs^ for all cases. As expected, *ρ*_w_^bs^ (Table S5) is lower than the standard density of 1 kg/L of water surrounding protein. It is well known that water weakens H-bonding leading to lower binding affinity than in vacuum. If this is true, then SPC/E model, for example, would provide the lowest binding affinity having the highest *ρ*_w_^bs^. However, this is not the case as this model provides the highest binding affinity (Table 4). In general, one has the strong correlation (correlation level *R* = 0.9) between *ρ*_w_^bs^ and Δ*G*_bind_ (Figure 8) that the higher water density is, the higher binding affinity is. Since this correlation is at odds with the role of water in weakening H-bonding, one expects that HBs alone do not govern ligand binding affinity.

Using parameters of water models (Table 1), one can show that Δ*G*_bind_ is not correlated with either the dielectric constant or dipole moment. Thus, one can not work out a unique factor that controls the binding affinity of Tamiflu to NA in AMBER. This is also true for other force fields.

#### 3.5.2. OPLS Force Field

As in the AMBER case, binding site volume (Figure 6), number of water molecules (Figure 7), and water density (Figure S10) do not show much variations among water models. In equilibrium the volume fluctuates around 5000 Å^3^  for all water models. SPC shows the highest water density, while the lowest value of *ρ*_w_^bs^ is given by TIP4P (Table S6). The latter model also has the smallest binding site. SPC/E and TIP3P have the same water density (Table S6) but different binding free energies (Table 4). For four water models there is a modest correlation (*R* = 0.67) between *ρ*_w_^bs^ and Δ*G*_bind_ (Figure 8). Again, similar to the AMBER case, this correlation cannot explain the binding affinity through the influence of water on HB network.

#### 3.5.3. CHARMM Force Field

The situation becomes entirely different in the case of CHARMM where the binding pocket volume (Figure 6) and number of water molecules inside it (Figure 7) are substantially higher than other force fields. TIP4P, which has the lowest volume in AMBER and OPLS, widens the pocket to the largest extent in CHARMM (Tables S5–S7). The number of water molecules in this model is about twofold larger than SPC/E. The packing of TIP4P water inside the binding site is also much denser (Figure S10 and Table S7) than other models having *ρ*_w_^bs^ ≈ 0.37 kg/L. Nevertheless, the corresponding binding free energy remains higher than SPC/E (Table 5). Overall, there is no correlation between *ρ*_w_^bs^ and Δ*G*_bind_ (Figure 8) in CHARMM.

#### 3.5.4. GROMOS Force Field

In GROMOS the binding pocket volume (Figure 6), the number of water molecules (Figure 7), and water density (Figure S10) are lower than other force fields (Table S8). This presumably comes from united-atom approximation. There is a pronounced difference in the binding free energies obtained by SPC and SPC/E due to different water densities. Thus, similar to AMBER and OPLS, Δ*G*_bind_ decreases with *ρ*_w_^bs^.

## 4. Conclusions

Our previous study [18] on binding affinity of Tamiflu to variants of influenza A/H5N1 neuraminidase has revealed that AMBER99SB is the best among popular force fields as it reproduces Δ*G*_bind_ with highest correlation and closest range with the experiments. In this paper, water models are tested with four force fields to examine their effects on the Tamiflu binding affinity toward the WT of A/H5N1 NA. As a result, TIP3P rather than SPC, SPC/E, and TIP4P goes along with AMBER99SB better than any combination of water models and other force fields.

Within one force field, in agreement with the GROMACS recommendation, combinations AMBER99SB-TIP3P, OPLS-TIP4P, CHARMM-TIP3P, and GROMOS-SPC are more suitable for simulation of ligand binding. Although this result has been obtained for NA-Tamiflu complex and its validity for other systems is a subject for further investigation, the choice of these combinations is recommended. One has to bear in mind that the above combinations may not work in some cases. For example, CHARMM-SPC is a better choice for WT NA than CHARMM-TIP3P. In the case of Y252H, SCP/E works better than other water models if one utilizes CHARMM (Table 7). We have demonstrated that within one force field the binding free energy greatly varies for different combination of force fields and water models. For WT NA SPC/E always provides the lowest binding free energy among all water models regardless of force fields.

Contrary to the remaining 3 force fields, Δ*G*_bind_ estimated by OPLS-AA/L force field does not vary much among water models (Table 5). Due to strong electrostatic interaction, their values are too low compared with other force fields and experiments. It remains unclear if this is an artifact of MM-PBSA approximation. Apolar solvation and entropy contributions are not affected by either force fields or water models (Tables 3–6), but other terms are sensitive to them. The HB network between Tamiflu and NA changes little upon water models in OPLS and AMBER force fields, while it does strongly in GROMOS. CHARMM is a medium case. The pronounced influence of aqueous models on water density inside binding pocket has been observed in CHARMM force field.

## Supplementary Material

Additional figures including the initial structure for MD simulation, the binding site, the time dependence of the number of hydrogen bonds between Os-eltamivir and receptor, typical snapshots of hydrogen bond networks, and time dependence of water density inside the binding pocket are presented. The tables on water density inside the binding site are provided.

## Figures and Tables

**Table 1:** Physical properties of water models [22]. All data is recorded at 25° and 1 atm. *q*_H_, *q*_O_, and *q*_L_ are the partial charges of hydrogen, oxygen, and lone pair, respectively. *θ* and *ϕ* are the H–O–H and lone pair-O–H angles, respectively. *ε* and *σ* are the well depth and vdW radius, respectively.

	*σ* (Å)	*ε* (kJ/mol)	*l*_1_ (Å)	*l*_2_	q_H_ (e)	q_O_/q_L_ (e)	*θ*	*ϕ*	Dipole moment	Dielectric constant
SPC	3.166	0.65	1		0.41	−0.82	109.47		2.27	65
SPC/E	3.166	0.65	1		0.423	−0.847	109.47		2.35	71
TIP3P	3.150	0.636	0.957		0.417	−0.834	104.52		2.35	82
TIP4P	3.153	0.648	0.957	0.15	0.52	−1.04	104.52	52.26	2.18	53
Exp.									2.95	78.4

**Table 2:** List of 50 residues surrounding the binding site.

Ile117	Arg118	Glu119	Leu134	Thr135	Gln136	Thr148	Val149	Lys150	Asp151
Arg152	Ser153	Arg156	Trp178	Ser179	Asp198	Asn221	Ile222	Leu223	Arg224
Thr225	Glu227	Pro245	Ser246	Glu276	Glu277	Arg292	Asp293	Asn294	Asn325
Pro326	Tyr347	Gly348	Val349	Lys350	Ser370	Arg371	Trp403	Ser404	Gly405
Tyr406	Ile427	Arg428	Gly429	Arg430	Pro431	Lys432	Thr439	Ser440	Gly441

**Table 3:** Binding free energies (in units of kcal/mol) of Tamiflu to WT of A/H5N1 NA calculated by MM-PBSA method and AMBER99SB force field with different water models.

	Δ*E*_elec_	Δ*E*_vdw_	Δ*G*_sur_	Δ*G*_PB_	−*T*Δ*S*	Δ*G*_bind_
SPC	− 181.16 ± 0.176	− 28.19 ± 0.029	−4.92 ± 0.005	183.09 ± 0.182	13.53	−17.65
SPC/E	− 203.02 ± 0.125	− 29.88 ± 0.035	− 5.17 ± 0.005	197.59 ± 0.189	15.32	−25.16
TIP3P	− 175.67 ± 0.176	− 28.98 ± 0.029	− 4.96 ± 0.005	181.50 ± 0.182	14.20	−13.91
TIP4P	− 173.92 ± 0.164	− 31.30 ± 0.031	− 5.10 ± 0.004	179.92 ± 0.187	13.68	−16.72

**Table 4:** The same as in Table 3 but for OPLS-AA/L force field.

	Δ*E*_elec_	Δ*E*_vdw_	Δ*G*_sur_	Δ*G*_PB_	−*T*Δ*S*	Δ*G*_bind_
SPC	− 211.98 ± 0.105	− 21.93 ± 0.011	− 4.99 ± 0.002	155.68 ± 0.077	14.23	−68.99
SPC/E	− 212.69 ± 0.107	− 21.60 ± 0.011	− 5.03 ± 0.002	155.01 ± 0.080	14.93	−69.38
TIP3P	− 213.83 ± 0.105	− 22.01 ± 0.011	− 4.93 ± 0.002	160.32 ± 0.080	15.25	−65.20
TIP4P	− 208.64 ± 0.105	− 20.37 ± 0.011	− 4.98 ± 0.002	153.48 ± 0.077	16.26	−64.25

**Table 5:** The same as in Table 3 but for CHARMM27 force field.

	Δ*E*_elec_	Δ*E*_vdw_	Δ*G*_sur_	Δ*G*_PB_	−*T*Δ*S*	Δ*G*_bind_
SPC	− 246.30 ± 0.429	− 12.93 ± 0.164	− 4.73 ± 0.005	230.70 ± 0.323	15.47	−17.79
SPC/E	− 260.11 ± 0.294	− 19.60 ± 0.154	− 4.94 ± 0.005	229.01 ± 0.181	14.79	−40.85
TIP3P	− 210.71 ± 0.429	− 17.82 ± 0.164	− 4.87 ± 0.005	195.15 ± 0.323	14.63	−23.62
TIP4P	− 198.60 ± 0.538	− 18.32 ± 0.186	− 4.29 ± 0.005	175.36 ± 0.369	14.79	−31.06

**Table 6:** The same as in Table 3 but for GROMOS96 43a1 force field.

	Δ*E*_elec_	Δ*E*_vdw_	Δ*G*_sur_	Δ*G*_PB_	−*T*Δ*S*	Δ*G*_bind_
SPC	− 116.24 ± 0.074	− 25.03 ± 0.014	− 4.22 ± 0.002	120.51 ± 0.074	13.19	**−11.79 **
SPC/E	− 147.34 ± 0.076	− 28.33 ± 0.013	− 4.72 ± 0.002	147.80 ± 0.076	14.03	**−18.56 **

**Table 7:** The binding free energy Δ*G*_bind_ (in kcal/mol) obtained by using the combination of CHARMM 27 with different water models for WT and mutants Y252H, N294S, and H274Y. The experimental results are taken from Collins et al. [23].

	SPC	SCP/E	TIP3P	TIP4P	Experiment
WT	−17.79	−40.85	−23.62	−31.6	−13.12
Y252H	−39.82	−11.41	−27.30	−31.69	−14.50
N294S	−33.14	−29.2	−23.02	−26.42	−10.48
H274Y	−30.26	−28.85	−17.06	−24.34	−9.77

## References

[1] Kollman PA, Massova I, Reyes C (2000). Calculating structures and free energies of complex molecules: combining molecular mechanics and continuum models. *Accounts of Chemical Research*.

[2] Kirkwood JG (1935). Statistical mechanics of fluid mixtures. *The Journal of Chemical Physics*.

[3] Aqvist J, Medina C, Samuelsson J-E (1994). A new method for predicting binding affinity in computer-aided drug design. *Protein Engineering*.

[4] Zwanzig RW (1954). High-temperature equation of state by a perturbation method. I. Nonpolar gases. *The Journal of Chemical Physics*.

[5] Karplus M, McCammon JA (2002). Molecular dynamics simulations of biomolecules. *Nature Structural Biology*.

[6] Lee FS, Chu Z-T, Bolger MB, Warshel A (1992). Calculations of antibody-antigen interactions: microscopic and semi-microscopic evaluation of the free energies of binding of phosphorylcholine analogs to McPC603. *Protein Engineering*.

[7] Bren U, Lah J, Bren M, Martnek V, Florin J (2010). DNA duplex stability: the role of preorganized electrostatics. * Journal of Physical Chemistry B*.

[8] Grubmüller H, Heymann B, Tavan P (1996). Ligand binding: molecular mechanics calculation of the streptavidin-biotin rupture force. *Science*.

[9] Mai BK, Viet MH, Li J MS (2010). Top leads for swine influenza A/H1N1 virus revealed by steered molecular dynamics approach. *Journal of Chemical Information and Modeling*.

[10] Berendsen JHC, Postma JPM, van Gunsteren WF, Hermans J, Pullmann B (1981). Interaction models for water in relation to protein hydration. *Intermolecular Forces*.

[11] Berendsen HJC, Grigera JR, Straatsma TP (1987). The missing term in effective pair potentials. *Journal of Physical Chemistry*.

[12] Jorgensen WL, Chandrasekhar J, Madura JD, Impey RW, Klein ML (1983). Comparison of simple potential functions for simulating liquid water. *The Journal of Chemical Physics*.

[13] Jorgensen WL, Madura JD (1985). Temperature and size dependence for Monte Carlo simulations of TIP4P water. *Molecular Physics*.

[14] Hess B, van der Vegt NFA (2006). Hydration thermodynamic properties of amino acid analogues: a systematic comparison of biomolecular force fields and water models. *Journal of Physical Chemistry B*.

[15] Florova P, Sklenovsky P, Banas P, Otyepka M (2010). Explicit water models affect the specific solvation and dynamics of unfolded peptides while the conformational behavior and flexibility of folded peptides remain intact. *Journal of Chemical Theory and Computation*.

[16] Hu Z, Jiang J (2009). Assessment of biomolecular force fields for molecular dynamics simulations in a protein crystal. *Journal of Computational Chemistry*.

[17] Almlof M, Brandsdal BO, Aqvist J (2004). Binding affinity prediction with different force fields: examination of the linear interaction energy method. *Journal of Computational Chemistry*.

[18] Nguyen TT, Mai BK, Li MS (2011). Study of tamiflu sensitivity to variants of A/H5N1 virus using different force fields. *Journal of Chemical Information and Modeling*.

[19] Cozzini P, Fornabaio M, Marabotti A, Abraham DJ, Kellogs GE, Mozzarelli A (2004). Free energy of ligand binding to protein: evaluation of the contribution of water molecules by computational methods. *Current Medicinal Chemistry*.

[20] Michel J, Tirado-Rives J, Jorgensen WL (2009). Prediction of the water content in protein binding sites. *Journal of Physical Chemistry B*.

[21] Wang L, Berne BJ, Friesner RA (2011). Ligand binding to protein-binding pockets with wet and dry regions. *Proceedings of the National Academy of Sciences of the United States of America*.

[22] Chaplin M Water structure and science. http://www.lsbu.ac.uk/water/.

[23] Collins PJ, Haire LF, Lin YP (2008). Crystal structures of oseltamivir-resistant influenza virus neuraminidase mutants. *Nature*.

[24] Hornak V, Abel R, Okur A, Strockbine B, Roitberg A, Simmerling C (2006). Comparison of multiple amber force fields and development of improved protein backbone parameters. *Proteins*.

[25] Brooks BR, Brooks CL, Mackerell AD (2009). CHARMM: the biomolecular simulation program. *Journal of Computational Chemistry*.

[26] Jorgensen WL, Tirado-Rives J (1988). The OPLS potential functions for proteins. Energy minimizations for crystals of cyclic peptides and crambin. *Journal of the American Chemical Society*.

[27] van Gunsteren WF, Billeter SR, Eising AA (1996). *Biomolecular Simulation: The GROMOS96 Manual and User Guide*.

[28] Webster RG, Govorkova EA (2006). H5N1 influenza—continuing evolution and spread. *The New England Journal of Medicine*.

[29] The Writing Committee of the World Health Organization (WHO) Consultation on Human Influenza A/H5 (2005). Avian influenza A (H5N1) infection in humans. *The New England Journal of Medicine*.

[30] Schrödinger (2010). *PyMOL: The PyMOL Molecular Graphics System, Version 1.3*.

[31] van Aalten DMF, Bywater R, Findlay JBC, Hendlich M, Hooft RWW, Vriend G (1996). PRODRG, a program for generating molecular topologies and unique molecular descriptors from coordinates of small molecules. *Journal of Computer-Aided Molecular Design*.

[32] Frisch MJ, Trucks GW, Schlegel HB (2004). *Gaussian 03, Revision C.02*.

[33] Silva AWSD, Vranken WF, Laue ED ACPYPE—AnteChamber PYthon Parser interfacE.

[34] Andre ASTR, Bruno ACH, Ricardo BA (2008). MKTOP: a program for automatic construction of molecular topologies. *Journal of the Brazilian Chemical Society*.

[35] Zoete V, Cuendet MA, Grosdidier A, Michielin O (2011). SwissParam: a fast force field generation tool for small organic molecules. *Journal of Computational Chemistry*.

[36] Guillot B (2002). A reappraisal of what we have learnt during three decades of computer simulations on water. *Journal of Molecular Liquids*.

[37] Bernal JD, Fowler RH (1933). A theory of water and ionic solution, with particular reference to hydrogen and hydroxyl ions. *The Journal of Chemical Physics*.

[38] Darden T, York D, Pedersen L (1993). Particle mesh Ewald: an N*·*log(N) method for Ewald sums in large systems. *The Journal of Chemical Physics*.

[39] Hockney RW, Goel SP, Eastwood JW (1974). Quiet high-resolution computer models of a plasma. *Journal of Computational Physics*.

[40] Berendsen HJC, Postma JPM, van Gunsteren WF, Dinola A, Haak JR (1984). Molecular dynamics with coupling to an external bath. *The Journal of Chemical Physics*.

[41] Parrinello M, Rahman A (1981). Polymorphic transitions in single crystals: a new molecular dynamics method. *Journal of Applied Physics*.

[42] Hess B, Kutzner C, van der Spoel D, Lindahl E (2008). GRGMACS 4: algorithms for highly efficient, load-balanced, and scalable molecular simulation. *Journal of Chemical Theory and Computation*.

[43] Cheng LS, Amaro RE, Xu D, Li WW, Arzberger PW, McCammon JA (2008). Ensemble-based virtual screening reveals potential novel antiviral compounds for avian influenza neuraminidase. *Journal of Medicinal Chemistry*.

[44] Barber CB, Dobkin DP, Huhdanpaa H (1996). The quickhull algorithm for convex hulls. *ACM Transactions on Mathematical Software*.

[45] Clarkson KL, Menlhorn K, Seidel R (1993). Four results on randomized incremental constructions. *Computational Geometry*.

[46] Chau PL (2004). Water movement during Ligand Unbinding from receptor site. *Biophysical Journal*.

[47] (2007). *MATLAB Version 7.0.1 (R2007a)*.

[48] Aruksakunwong O, Malaisree M, Decha P (2007). On the lower susceptibility of oseltamivir to influenza neuraminidase subtype N1 than those in N2 and N9. *Biophysical Journal*.

