# Supplementary material for: Effects of Water Models on Binding Affinity: Evidence from All-Atom Simulation of Binding of Tamiflu to A/H5N1 Neuraminidase
Source: ScientificWorldJournal. 2014 Feb 2;2014:536084. doi: 10.1155/2014/536084 (PMC3929574; doi:10.1155/2014/536084)
Supplement: Supplementary file 1 — Additional figures including the initial structure for MD simulation, the binding site, the time dependence of the number of hydrogen bonds between Os-eltamivir and receptor, typical snapshots of hydrogen bond networks, and time dependence of water density inside the binding pocket are presented. The tables on water density inside the binding site are provided. [file 536084.f1.pdf]

Supporting information for:

Effects of water models on binding affinity: Evidences from all-atom simulation of binding of Tamiflu to A/H5N1 neuraminidase

Trang Truc Nguyen,[†] Man Hoang Viet,[‡] and Mai Suan Li[*,‡]

Institute for Computational Science and Technology, Quarter 6, Linh Trung Ward, Thu Duc District, Ho Chi Minh City, Vietnam, and Institute of Physics, Polish Academy of Sciences, Al. Lotnikow 32/46, 02-668 Warsaw, Poland

E-mail: masli@ifpan.edu.pl

[*]To whom correspondence should be addressed
[†]Institute for Computational Science and Technology
[‡]Institute of Physics

Table S1: Binding free energy (in units of kcal/mol) of Tamiflu to WT and three mutations of A/H5N1 calculated by MM-PBSA method and CHARMM27 force field with SPC water model.

**SPC**	ΔE_{elec}	ΔE_{vdw}	ΔE_{sur}	ΔG_{PB}	$-T\Delta S$	ΔG_{bind}
WT	-246.30	-12.93	-4.73	230.70	15.47	-17.79
Y252H	-256.21	-19.51	-4.98	224.05	16.83	-39/82
N294S	-258.37	-16.77	-4.92	231.71	15.21	-33.14
H274Y	-264.47	-17.23	-4.93	239.92	16.45	-30.26

Table S2: Binding free energy (in units of kcal/mol) of Tamiflu to WT and three mutations of A/H5N1 calculated by MM-PBSA method and CHARMM27 force field with SPC/E water model.

**SPC/E**	ΔE_{elec}	ΔE_{vdw}	ΔE_{sur}	ΔG_{PB}	$-T\Delta S$	ΔG_{bind}
WT	-260.11	-19.60	-4.94	229.01	14.79	-40.85
Y252H	-176.32	-21.50	-4.59	175.53	15.47	-11.41
N294S	-247.28	-18.03	-5.14	225.61	15.64	-29.20
H274Y	-264.78	-19.79	-4.69	244.35	16.06	-28.85

Table S3: Binding free energy (in units of kcal/mol) of Tamiflu to WT and three mutations of A/H5N1 calculated by MM-PBSA method and CHARMM27 force field with TIP3 water models.

**TIP3**	ΔE_{elec}	ΔE_{vdw}	ΔE_{sur}	ΔG_{PB}	$-T\Delta S$	ΔG_{bind}
WT	-210.71	-17.82	-4.87	195.15	14.63	-23.62
Y252H	-218.97	-18.68	-5.01	201.07	14.29	-27.30
N294S	-215.56	-17.00	-5.21	199.19	15.56	-23.02
H274Y	-229.96	-17.75	-4.93	220.82	14.76	-17.06

Table S4: Binding free energy (in units of kcal/mol) of Tamiflu to WT and three mutations of A/H5N1 calculated by MM-PBSA method and CHARMM27 force field with TIP4 water model.

**TIP4**	ΔE_{elec}	ΔE_{vdw}	ΔE_{sur}	ΔG_{PB}	$-T\Delta S$	ΔG_{bind}
WT	-198.60	-18.32	-4.29	175.36	14.79	-31.06
Y252H	-249.00	-18.88	-4.92	225.93	15.18	-31.69
N294S	-171.57	-17.69	-4.31	151.41	15.74	-26.42
H274Y	-253.02	-15.97	-4.83	233.23	16.25	-24.34

Table S5: Volume site and density of water molecules at the binding site for all water models in AMBER force field. The results were averaged over snapshots collected in equilibrium.

AMBER	ΔG_{bind}	$\Delta E_{elec} + \Delta G_{PB}$	Volume site ($Å^3$)	Number of water	Density of water (kg/l)
SPC	-17.65	1.93	5276.96	43	0.241
SPC/E	-25.16	-5.43	5351.87	46	0.256
TIP3P	-13.91	5.83	5260.49	42	0.239
TIP4P	-16.72	6.00	5134.76	43	0.248

Table S6: The same as on Table S5 but in OPLS force field

OPLS	ΔG_{bind}	$\Delta E_{elec} + \Delta G_{PB}$	Volume site ($Å^3$)	Number of water	Density of water (kg/l)
SPC	-68.79	-56.30	5304.67	39	0.218
SPC/E	-69.38	-57.68	5074.72	34	0.200
TIP3P	-65.20	-53.51	5166.72	35	0.200
TIP4P	-64.25	-55.16	4968.40	31	0.189

Table S7: The same as on Table S5 but in CHARMM force field

CHARMM	ΔG_{bind}	$\Delta E_{elec} + \Delta G_{PB}$	Volume site ($Å^3$)	Number of water	Density of water (kg/l)
SPC	-17.79	-15.60	6034.13	52	0.258
SPC/E	-40.85	-31.10	5482.91	42	0.227
TIP3P	-23.62	-15.56	5851.24	50	0.254
TIP4P	-31.06	-23.24	6846.50	85	0.370

Table S8: The same as on Table S5 but in GROMOS force field

GROMOS	ΔG_{bind}	$\Delta E_{elec} + \Delta G_{PB}$	Volume site (Å^3)	Number of water	Density of water (kg/l)
SPC	-11.79	4.27	4602.90	25	0.162
SPC/E	-18.56	0.46	4841.46	29	0.179

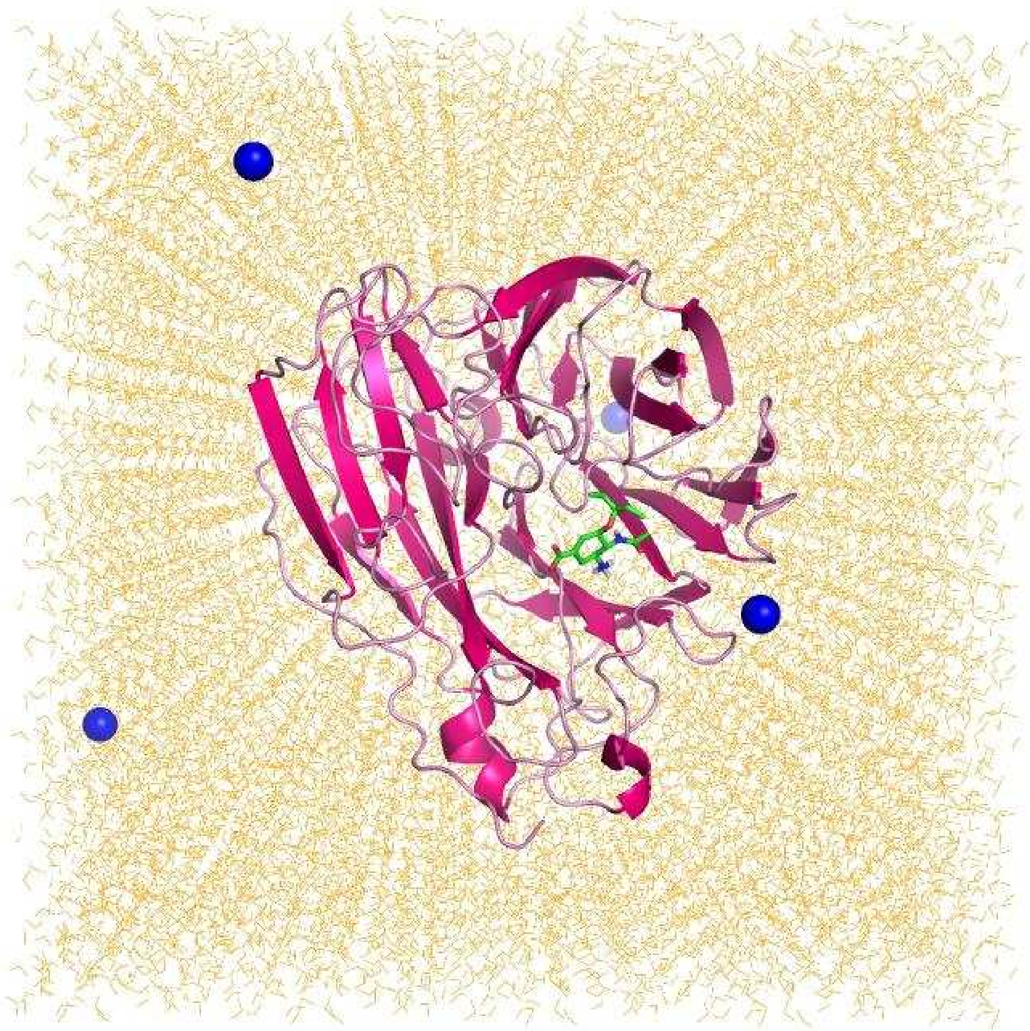

Figure S1: Initial structure for MD runs of Tamiflu and NA. Tamiflu is colored in green, while blue spheres are ions Na^{+} added to neutralize the system.

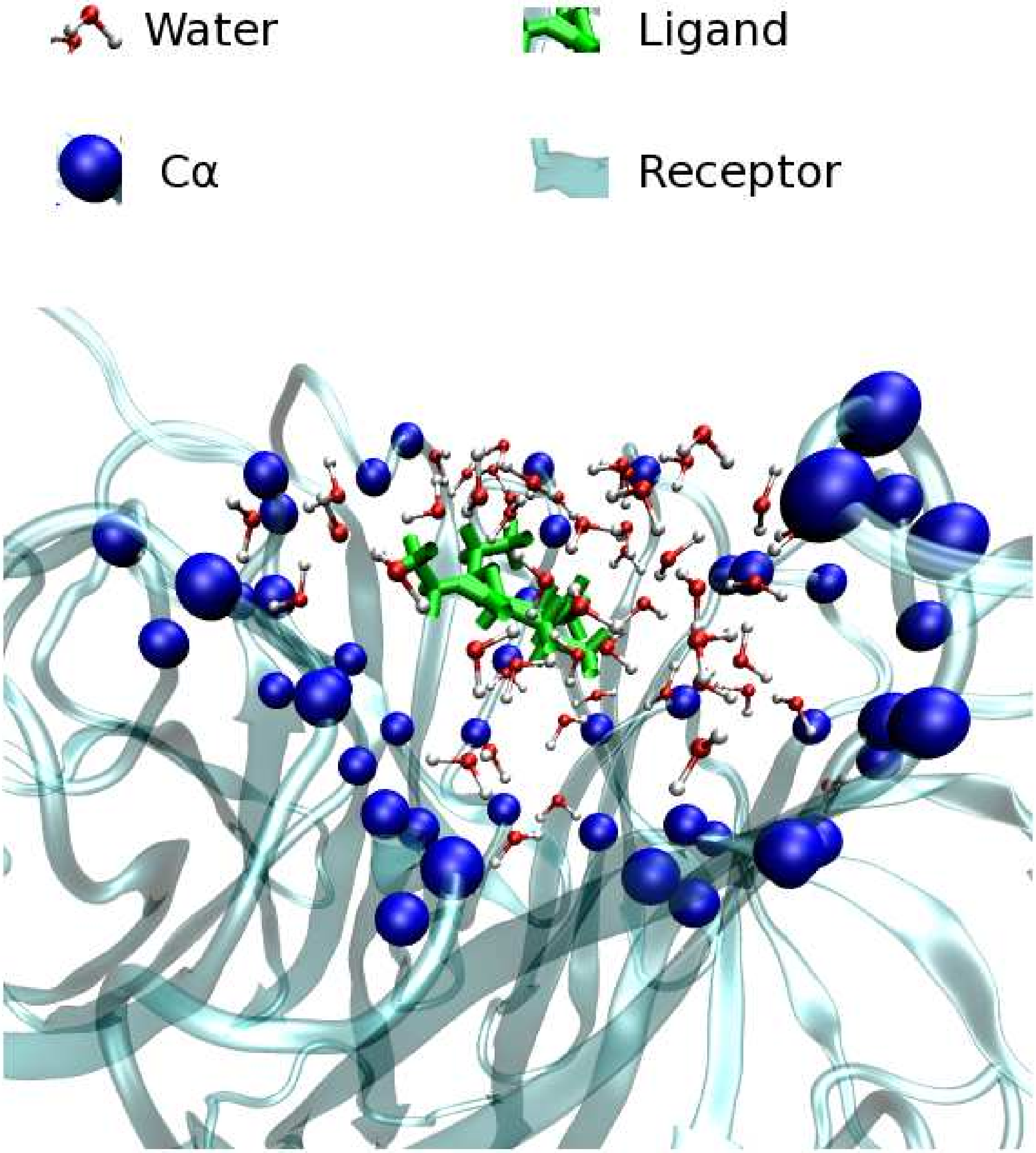

Figure S2: Binding site with water molecules (red and white) and Tamiflu (green). C_α-atoms of fifty residues which define the binding pocket are shown in blue ball.

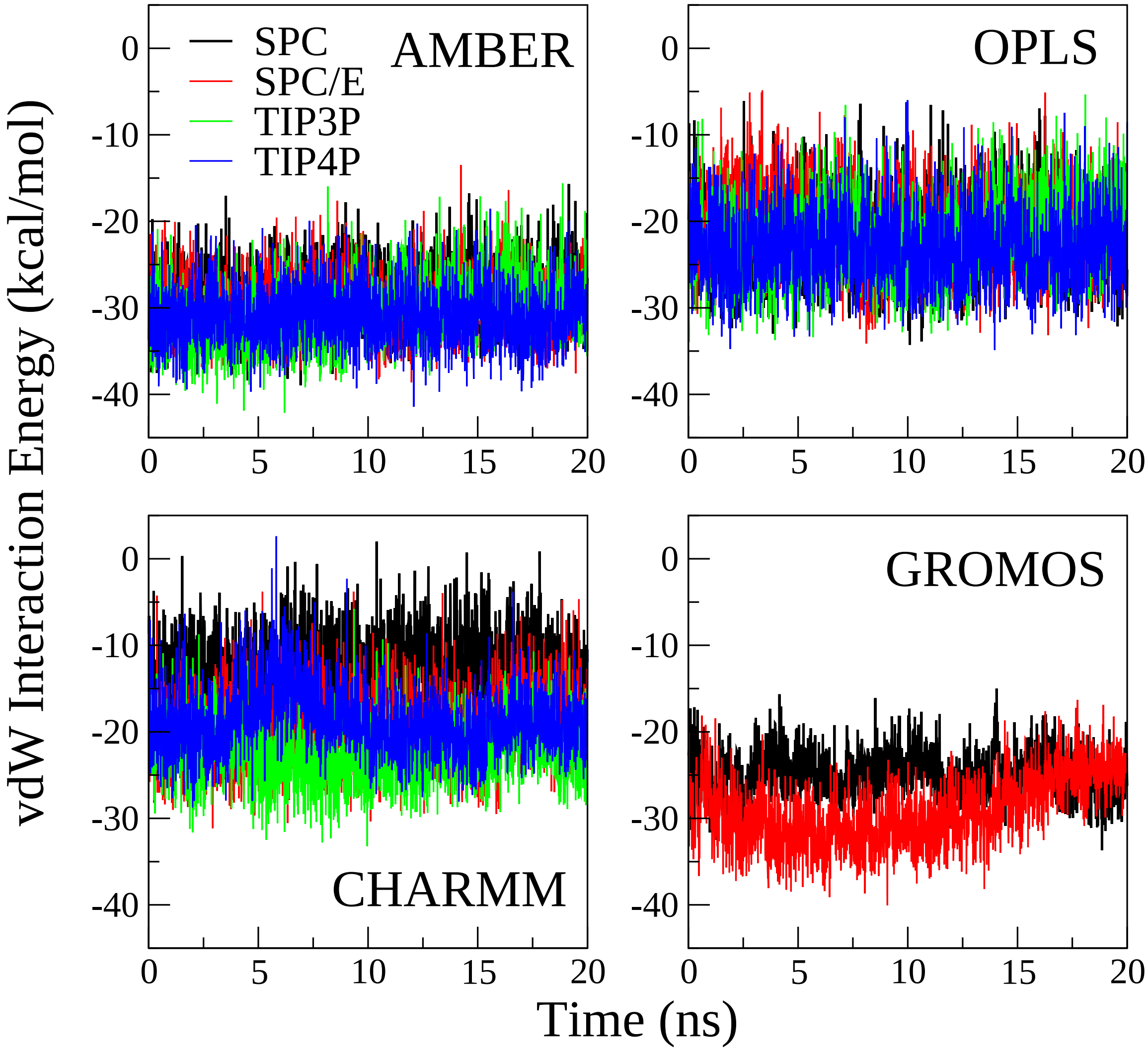

Figure S3: Time dependence of vdW interaction energies of NA with Tamiflu for different combinations of force fields and water models.

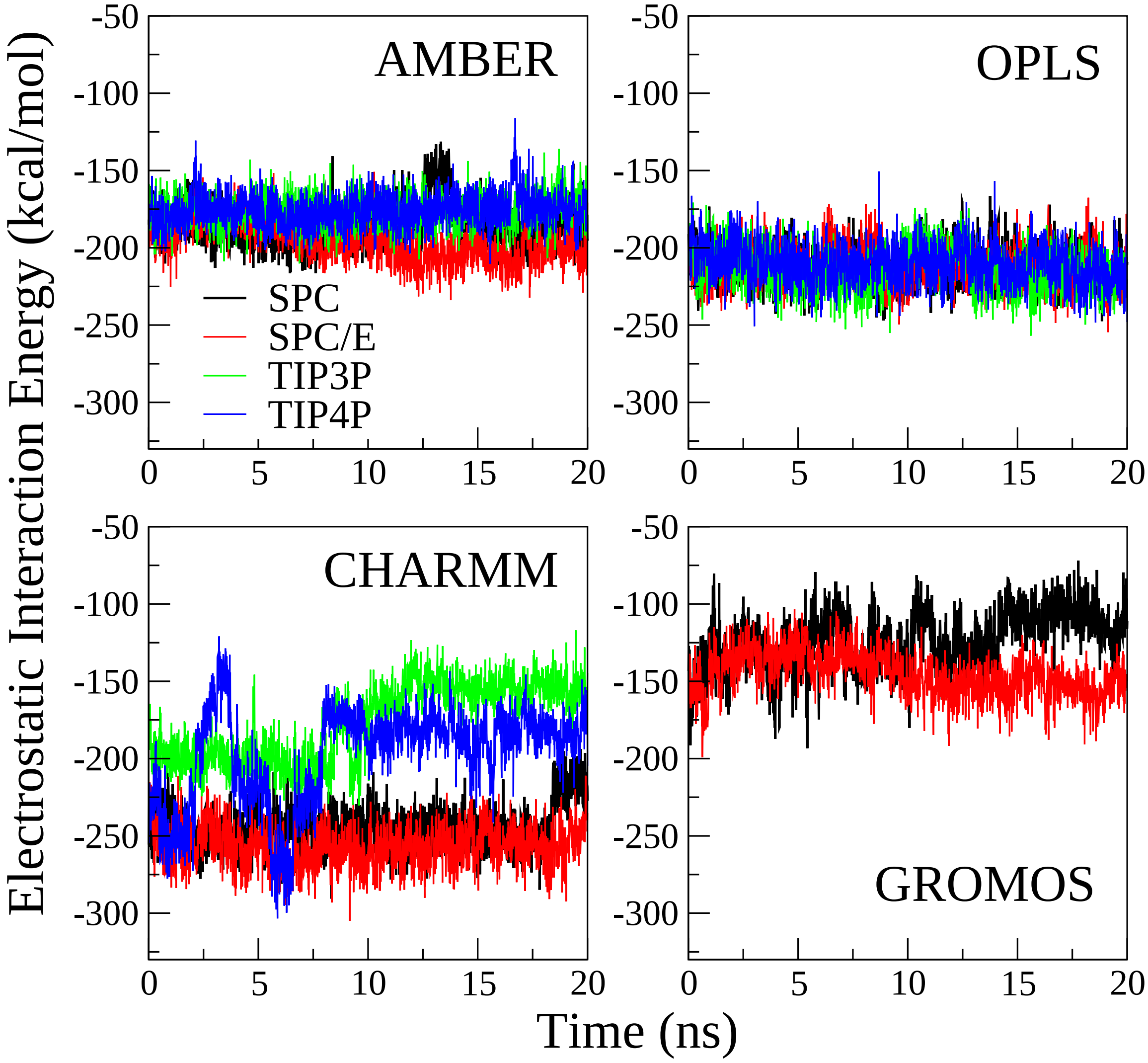

Figure S4: Time dependence of electrostatic interaction energies between NA and Tamiflu for different sets of force fields and water models.

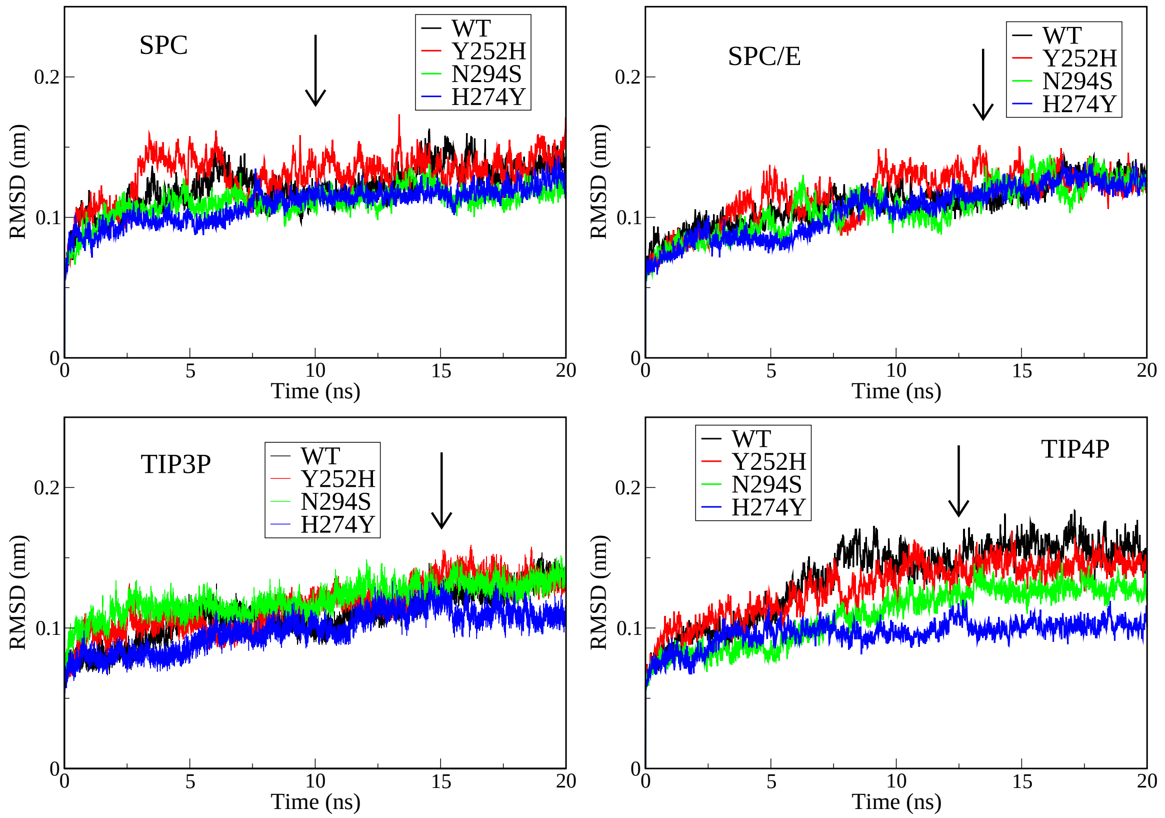

Figure S5: Time dependence of RMSD of WT and mutants of NA for different combinations of CHARMM 27 and water models. Arrows roughly refer to time when the system reaches equilibrium. Snapshots collected in the equilibrium were used to estimate the binding free energy by the MM-PBSA method.

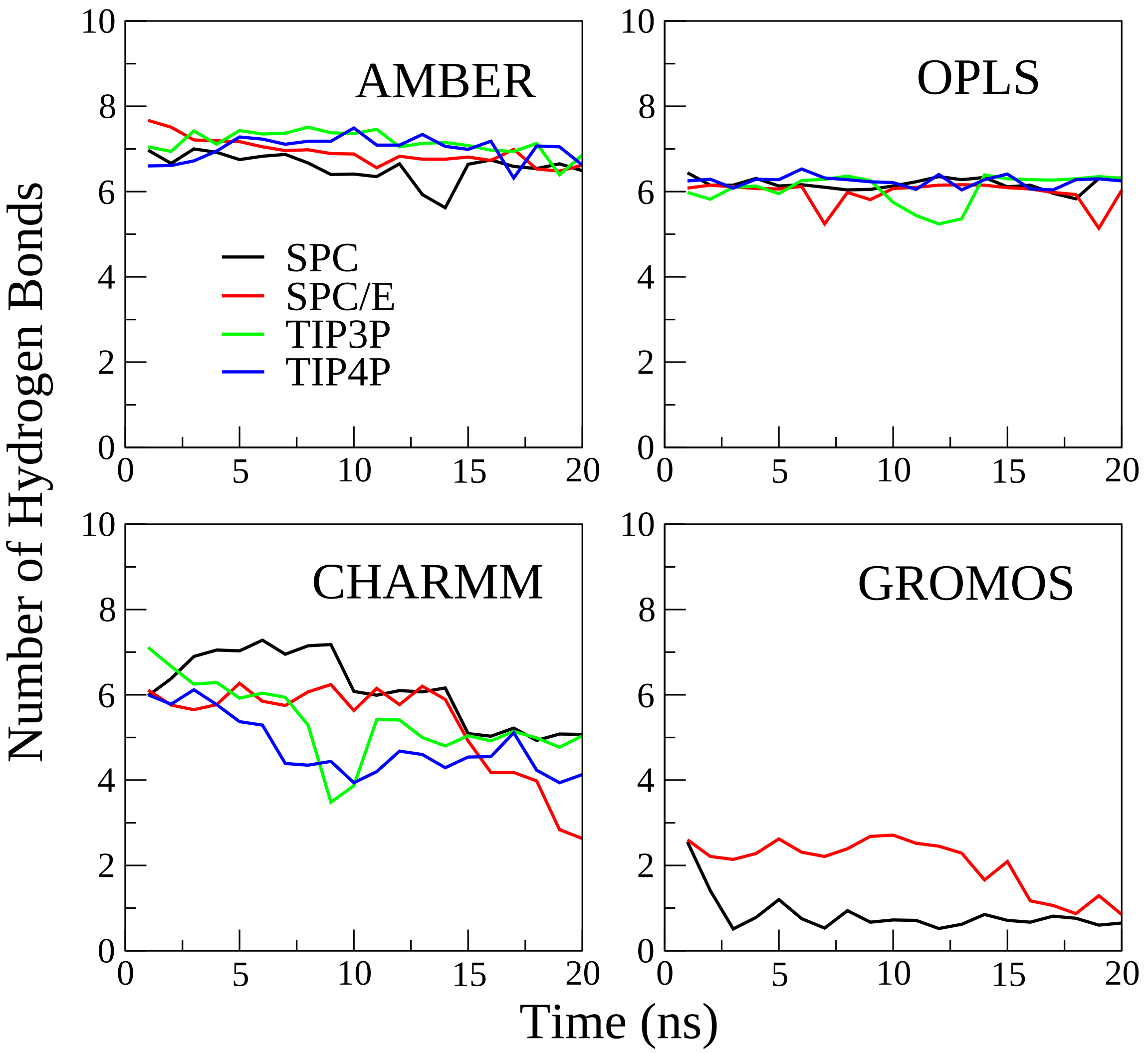

Figure S6: Time dependence of the number of HBs between NA and Tamiflu for different sets of force fields and water models. In equilirium the average number of HBs $\overline{H(t)} = 6.6(6.2, 5.5, 0.7), 6.7(6.0, 4.7, 1.6), 7.0(6.0, 5.1)$ and 7.0 (6.2, 4.4) for SPC, SPC/E, TIP3P, TIP4P, respectively. Numbers outside parenthesis refer to AMBER, while those in parenthesis are for OPLS, CHARMM and GROMOS, respectively.

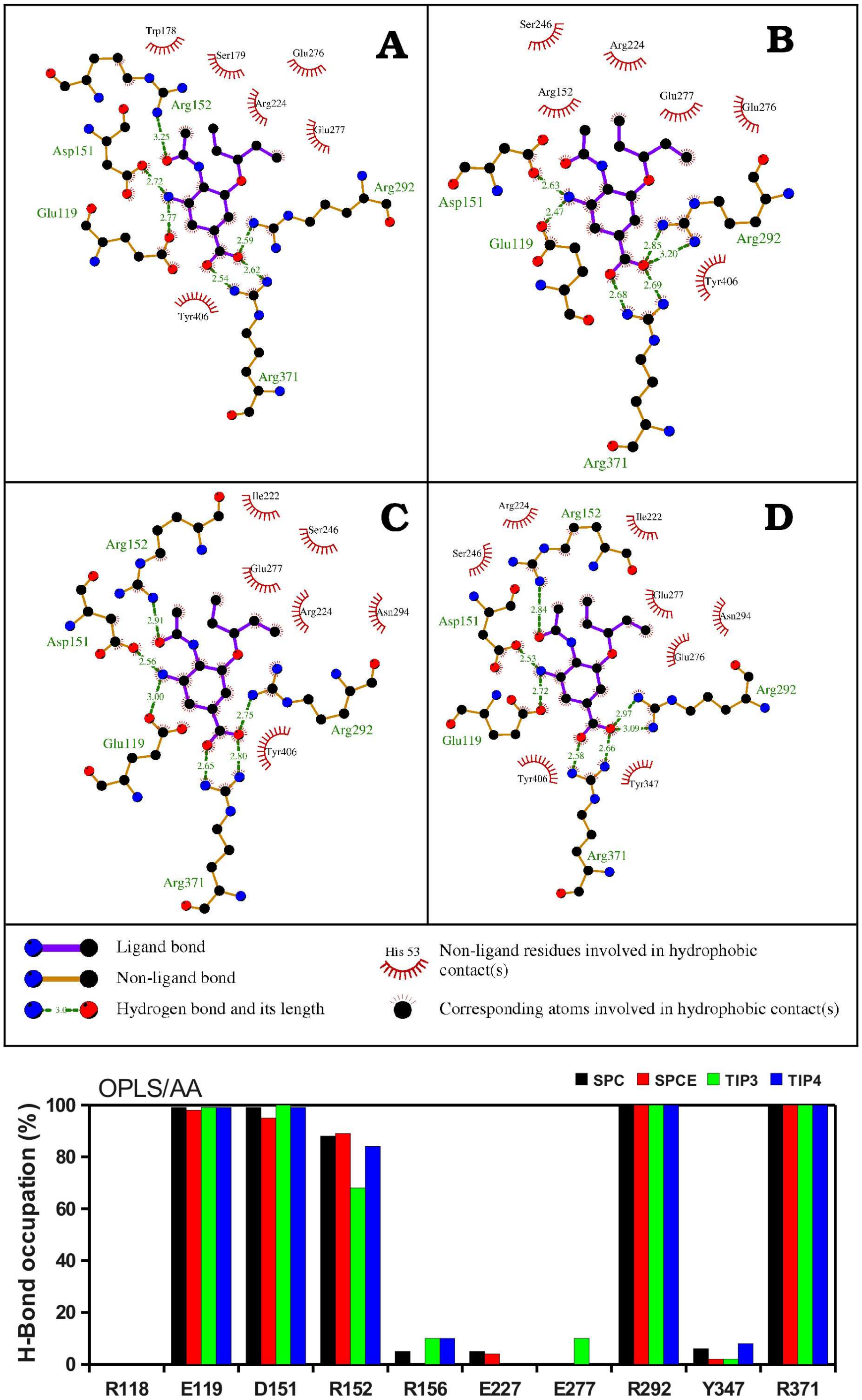

Figure S7: Typical snapshots for hydrogen bond network between Tamiflu's charged groups and residues of NA at the binding site obtained by OPLS force field with SPC (A), SPC/E (B), TIP3P (C) and TIP4P (D). HB networks are formed with $-COO^-$ and $-NH_2$ $(R371, R292)$, $-OH$ $(Y347)$; $-NH3^+$ and $-COO^-(D151, E119)$; NHAc and $-NH_2$ $(R152)$ of the receptor. All hydrogen atoms are implicit. The lower panel refers to the probability of formation of HBs between Tamiflu and NA. The results are averaged over the last 2ns of simulation. Black, red, green and blue refer to SPC, SPC/E, TIP3P and TIP4P, respectively

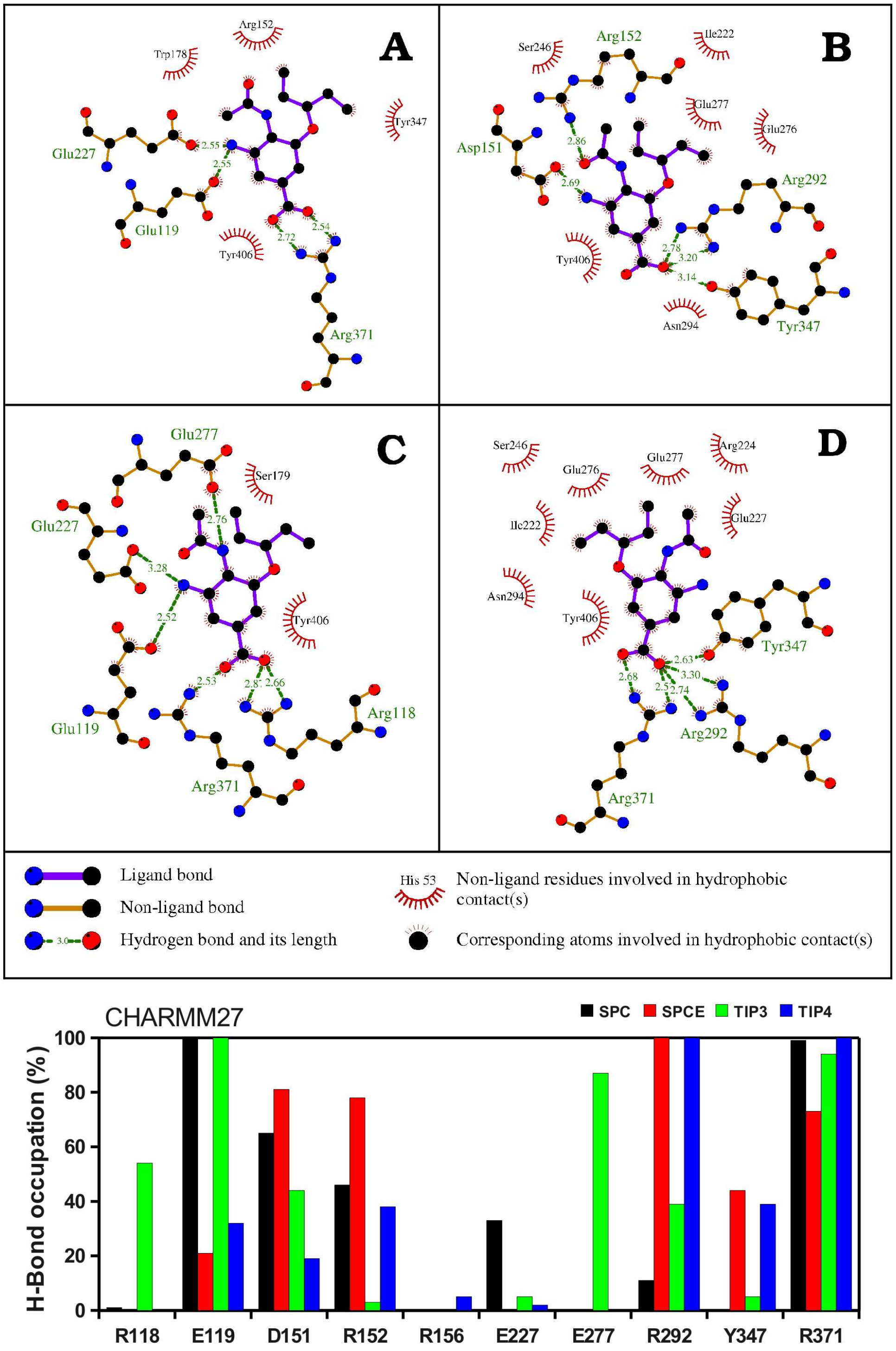

Figure S8: The same is in Fig. S7 but for CHARMM27 force field. Hydrogen bonds are as follows. (A) SPC: $-COO^-$ and $-NH_2$ $(R371)$, $-NH3^+$ and $-COO^-(E227,E119)$; (B) SPC/E: $-COO^-$ and $-NH_2$ $(R292,Y347)$; $-NH3^+$ and $-COO^-$ $(D151)$; NHAc and $-NH_2$ $(R152)$. (C) TIP3P: $-COO^-$ and NH_2 $(R118,R371)$; $-NH3^+$ and $-COO^-(E119,E227)$, NHAc and $-COO^-(E277)$. (D) TIP4P: $-COO^-$ and NH_2 $(R371,R292)$, $-OH$ $(Y347)$. All hydrogen atoms are implicit. The lower panel refers to the probability of formation of HBs between Tamiflu and WT.

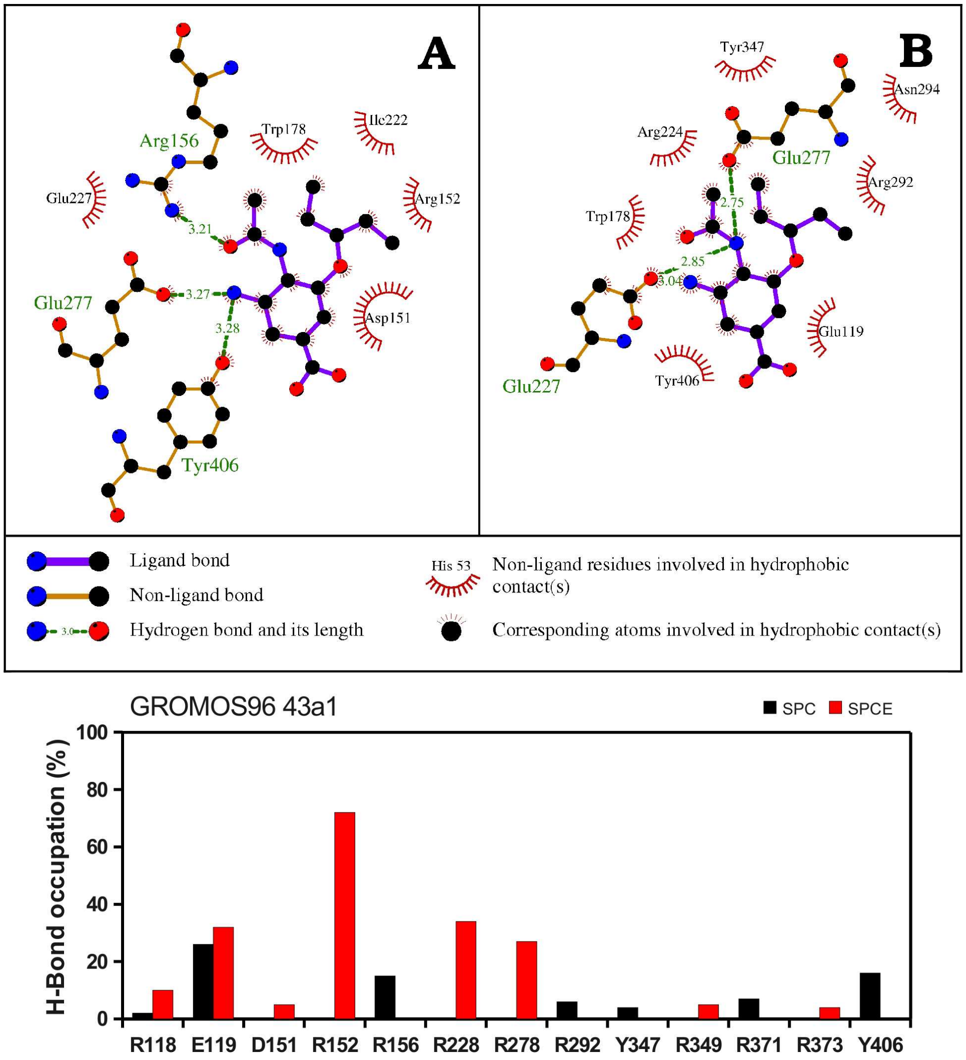

Figure S9: The same is Fig. S7 but for GROMOS96 43a1 force field. Hydrogen bonds are as follows. (A) SPC: $-NH3^+$ and $-COO^-(E277)$, $-OH$ $(Y347)$; NHAc and $-COO^-(R156)$ (B) SPC/E: $-NH3^+$ and $-COO^-$ $(E227)$; NHAc and $-COO^-$ $(E227)$, $-COO^-$ $(E277)$. All hydrogen atoms are implicit. The lower panel refers to the probability of formation of HBs between Tamiflu and WT. The results are averaged over the last 2ns of simulation. Black and red refer to SPC, SPC/E, respectively.

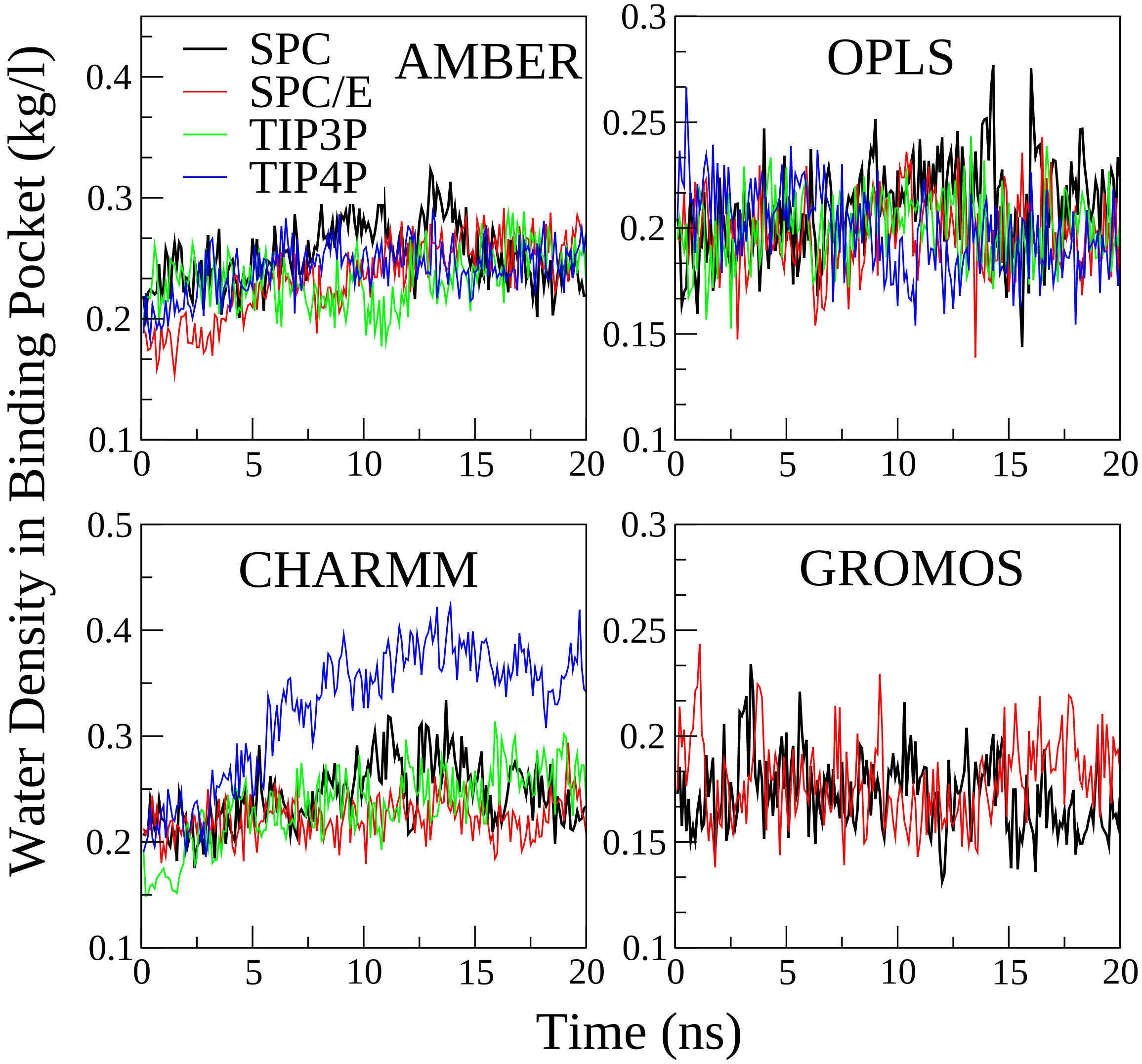

Figure S10: Time dependence of water density inside the binding pocket for different force fields and water models.
